# High-performance flexible supercapacitors based on electrochemically tailored three-dimensional reduced graphene oxide networks

**DOI:** 10.1038/s41598-017-18593-3

**Published:** 2018-01-12

**Authors:** Taniya Purkait, Guneet Singh, Dinesh Kumar, Mandeep Singh, Ramendra Sundar Dey

**Affiliations:** Institute of Nano Science and Technology (INST), Mohali, 160062 Punjab India

## Abstract

A simple approach for growing porous electrochemically reduced graphene oxide (pErGO) networks on copper wire, modified with galvanostatically deposited copper foam is demonstrated. The as-prepared pErGO networks on the copper wire are directly used to fabricate solid-state supercapacitor. The pErGO-based supercapacitor can deliver a specific capacitance (C_sp_) as high as 81±3 F g^−1^ at 0.5 A g^−1^ with polyvinyl alcohol/H_3_PO_4_ gel electrolyte. The C_sp_ per unit length and area are calculated as 40.5 mF cm^−1^ and 283.5 mF cm^−2^, respectively. The shape of the voltammogram retained up to high scan rate of 100 V s^−1^. The pErGO-based supercapacitor device exhibits noticeably high charge-discharge cycling stability, with 94.5% C_sp_ retained even after 5000 cycles at 5 A g^−1^. Nominal change in the specific capacitance, as well as the shape of the voltammogram, is observed at different bending angles of the device even after 5000 cycles. The highest energy density of 11.25 W h kg^−1^ and the highest power density of 5 kW kg^−1^ are also achieved with this device. The wire-based supercapacitor is scalable and highly flexible, which can be assembled with/without a flexible substrate in different geometries and bending angles for illustrating promising use in smart textile and wearable device.

## Introduction

Supercapacitors, an exclusive class of energy storage devices can look beyond the privileges of rechargeable batteries regarding instant power delivery and ability to sustain millions of charge-discharge cycles at higher current densities^1,2^. Unlike batteries, where, energy storage occurs by means of redox reactions; supercapacitors, particularly electrochemical double layer capacitors (EDLCs) store opposite charges at the interface of active layer/electrolyte only by physisorption, and their fast ion-exchange kinetics makes them specifically appealing to be used in high power applications such as hybrid electrical vehicles and power stations^3^. Integrating storage advantage with rate performances seems to be the solution to the recent demands in compact, multifunction portable electronic equipment. Miniature devices having wire/fiber based supercapacitors (WSCs/FSCs) as energy storage systems are thus earning prolific interests due to their versatile functionalities including high flexibility, foldability, and light-weight power sources^4–6^. Performance, design as well as mechanical properties of the WSC/FSCs are also critical for the evolution of modern smart devices. WSCs with high power density and long lifetime offer new vistas to the researchers^7–9^. Their electrochemical performances depend on wire current collectors, active materials, gel electrolytes and device structures^10^. Various types of wires/fibers as current collectors such as metal wire^11^, metal alloy^8^, gold coated plastic wires^12,13^, nylon fiber^14^, carbon fiber^6^, carbon nanotubes (CNTs) and graphene fibers^15–18^ have been used till date into FSCs/WSCs. The performance of WSC not only depends on the wire/fiber core of the device but also is governed by the porosity and surface area of the active materials modified on conductive wire core. This can only be partially achieved by using nanostructured materials high surface-to-volume ratio. Current research interest is focused on increasing the energy density of WSCs without sacrificing the device performances in terms of power density and cycle life^4^. Ionic diffusion resistance through the pores and lower electron-transfer resistance between active material and current collector has significant role to improve the rate performance of solid-state WSCs^19^. Binder-free solid-state WSC is thus receiving increasing attention to accomplish the rising needs of the essential features of an energy storage device.

Graphene sheets are extensively looked upon as a promising electrode material because of their ultra-high surface area and excellent conductivity as well as high mechanical and chemical stability, low-cost and large-scale production^20^,^21^,^22^. Recently, self-assembled three-dimensional (3D) graphene structures have been widely recognized as active electrode materials as it possesses striking features including highly-exposed surface areas, high electrical conductivity, and excellent chemical stability which are the essential features for supercapacitor applications^23^. Therefore, for the construction of new porous 3D architecture with high stability, porous reduced graphene oxide (rGO) is perhaps the best choice as electrode material for supercapacitor^23^. However, as 3DrGO is mechanically fragile and to transfer the 3D architecture to a current collector is really a substantial challenge, flexible device fabrication with 3DrGO suffers from conductivity issues and thus poor electrochemical performances. As a result, low specific capacitance, as well as energy density, resulted from the device^18,24^. In our previous work, we have shown an easy and low-cost strategy to prepare self-assembled 3DrGO on copper foam (Cuf), for the cost-effective fabrication of supercapacitor electrodes in aqueous solution^23^. Three-dimensional architecture of rGO on the Cu-based electrode are not studied well, although there are some reports related to Cu supported graphene material as electrodes, the performances of the active material as well as device properties at solid-state needs to be explored^25^. Recently, some reports have been made on producing electrochemically reduced conductive rGO via electrophoretic deposition method^26,27^. This process requires very high potential (3–30 V) and reaction time (15–30 min), to produce thin films with very less porosity, which is not suitable for energy storage applications. The electrochemically deposited porous reduced graphene oxide (pErGO) networks via bulk electrolysis over Cu wire electrode have great importance due to the high conductivity of porous graphene and low barrier resistance between Cu and graphene. The pores of the three-dimensional network are fully exposed to the electrolyte for the access of the ions in the double-layer formed between electrode and electrolyte. Furthermore, pErGO networks not only reduce the ion diffusion resistance but also improve the electronic conductivity, results to high rate performance of the wire-shaped supercapacitor device.

In last few years some reports have come on carbon-based wire-shaped supercapacitor^15,18,28–35^. Qu and co-workers have developed all graphene core-sheath flexible supercapacitor, where 3D graphene-like structure was synthesized on graphene fiber^18^. Lamberti *et al*. have reported graphene aerogel on Cu wire, synthesized by a hydrothermal method for wire-based supercapacitor^33^. This method is not sufficient to produce high-quality graphene, and there is a chance for oxidation of copper in the reaction condition, thus suffering from interfacial barrier resistance. Some complicated procedures and challenging synthesis routes have been employed to produce porous graphene for wire-shaped supercapacitor, resulting in a poor performance of the device. Therefore, all the previously reported carbon-based WSCs have suffered from impractical specific capacitance and low energy density. Challenges are still open to fabricating high-performance WSC with a straightforward and economical method for producing bulk-scale active electrode material. Herein, we have explored a one-step and cost-effective electrochemical approach for producing high-quality porous rGO networks on Cuf/Cu wire electrode. Potentially, electrochemical reduction of graphene-oxide is the most convenient, least-expensive, time-saving and easy to scale-up to the industrial scale method which produces highly controllable, conformal films without the need for volatile solvents or reducing agents^36^. The pErGO-based electrodes were assembled on the flexible sheet in a planar configuration with an ionogel as an electrolyte as well as a separator. The *in situ* grown porous graphene networks in co-axial configuration allow increasing the available surface area exposed to the electrolyte^24^. The combination of large-scale production of porous 3D graphene by the electrochemical approach and high electrochemical performance of porous graphene on Cu wire paves-the-way for wearable and textile application.

## Experimental

### Modification of copper foam on copper wire

Cuf thin film was grown on commercially available Cu wire by galvanostatic electrodeposition method mentioned in our previous report^23^. In a typical preparation procedure, a piece of highly pure and cleaned Cu wire (with an active area of 0.29 cm^2^) was used as a substrate (cathode) for the deposition of Cuf and another piece of Cu foil used as a counter electrode (anode). All Cu substrates were cleaned by dilute HNO_3_ followed by water and ethanol. They were finally washed with Millipore water repeatedly and stored in argon atmosphere when not in use to avoid atmospheric oxidation^37^. A constant current density (e.g. 1.5 A cm^−2^) was applied to cathode and anode in an electrochemical cell containing 0.4 M CuSO_4_ and 1.5 M H_2_SO_4_ for 45 s. The distance between anode and cathode was fixed at 1 cm. The formation process of the galvanostatic electrodeposition of copper foam can be mechanistically described as a reason of hydrogen gas evolution. In this process, hydrogen bubbles play a crucial role in the formation of the porous structure^38^. Deposition of copper to the substrate and hydrogen evolution from the substrate occurs simultaneously. As a result, a continuous path formation (generation of porosity) occurs from the substrate to the electrolyte-air interface. Deposition of metals will not take place where bubbles are present, because, no metal ions are available there^37^. Thus, the numerous hydrogen bubbles evolved at different locations on the substrate generate the pores within the deposited metal. The resulting structures are 3D free-standing foams of copper.

### Electrochemical deposition of pErGO on Cuf

**Figure 1:**
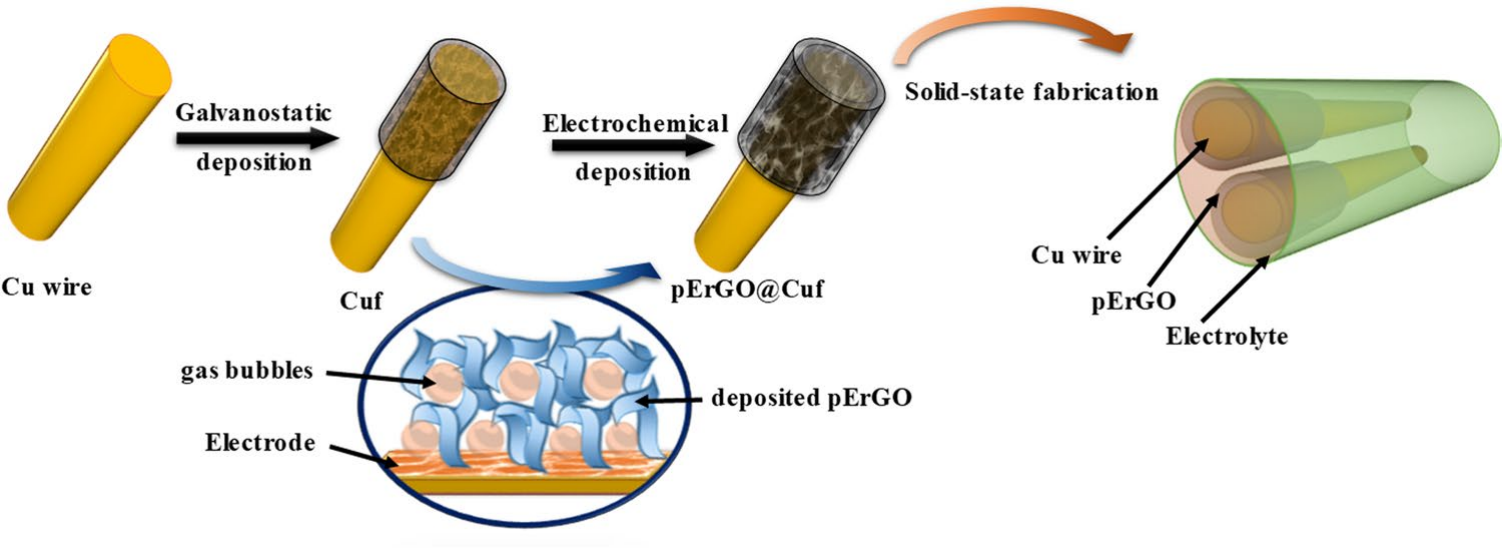
Schematic representation of the overall design and process flow for the stepwise fabrication of 3DrGO@Cuf/Cu-wire supercapacitor.

Reduced graphene-oxide networks were electrochemically grown by electrolysis of GO solution with the Cuf modified Cu-wire electrodes (as shown in Fig. 1). Electrolysis was carried out in four different concentrations (1, 3, 5 and 7 mg mL^−1^) of GO aqueous suspension containing 1 M phosphoric acid solution at an applied potential of −1.2 V against Saturated Calomel Electrode (SCE) for 200 seconds^24^. The optimum GO concentration for attaining maximum specific capacitance (C_sp_) was found to be 5 mg mL^−1^ (Figure S1, supporting information). During the electrochemical process, GO sheets were reduced under constant potential into conductive ErGO as reported in the literature^24,39^. To ensure the complete reduction of the electrodeposited pErGO layer on the electrodes, 20 potential cycles were carried out in the presence of 1 M phosphoric acid solution. Electro-reduction of oxygen-containing groups in GO leads to conductive rGO sheets in the form of three-dimensional interpenetrating networks with a thickness of about 30 µm (Figure S2, supplementary information) on Cuf structure under the influence of the applied electric field. The well-distributed Cuf pores allow for deposition of rGO in and across it and exposure to the electrolyte for the complete access of ions to form electrochemical double layers. The mass of the active material (pErGO) was measured by weighing the modified wire before and after rGO deposition by a high precision laboratory balance. For comparison, GO aqueous suspension was dropcast on Cuf, supported Cu substrate and freeze-dried for overnight. Electrochemical reduction (50 cycles) was then performed in 1 M PBS electrolyte to reduce the dropcast GO to rGO.

### Fabrication of solid-state flexible symmetrical supercapacitor

Stepwise fabrication of all-solid-state supercapacitor has been demonstrated in Fig. 1. Two identical pErGO@Cuf/Cu electrodes were assembled in a planar configuration on polyethylene terephthalate (PET) sheet with PVA/H_3_PO_4_ gel electrolyte. Briefly, the electrodes were immersed in PVA/H_2_O gel containing H_3_PO_4_ as the electrolyte and arranged in parallel geometry on a flexible platform (like PET) for drying. While choosing the solid-state electrolyte, the most important criterion was that the electrolyte must be ionically conducting but electrically neutral. An aqueous gel electrolyte of PVA/H_3_PO_4_ perfectly fits the need. The versatility of Cu is fully explored here as it can act as an integrated current collector as well as the stable Cuf developed on it can serve as a template for pErGO deposition without the need of any external organic toxic material as a binder. For the preparation of the gel electrolyte, typically 1 g of PVA was added to 10 mL of water and warmed to 80 °C until the solution is clear i.e. the gel is entirely soluble in the water. Stirring was continued for another 30 minutes after the addition of 1 M H_3_PO_4_ to prepare the solid-state gel electrolyte. Here PVA gel adds to the flexibility and separates the electrodes from any danger of undesirable short-circuits^18^. The as-fabricated supercapacitor device was properly dried before testing.

## Results and Discussions

### Proposed mechanism for the formation of pErGO materials

Electrodeposition of pErGO directly from an aqueous suspension of GO simplify the process and produces highly controllable and conformal films. It is well accepted that the electrochemically grown rGO sheets are more hydrophobic, have weaker electrostatic repulsion and stronger π−π stacking interaction than those of their GO precursors^39^. Thus, they were self-assembled to form 3D interpenetrating networks and deposited onto the substrate electrode upon the driving of electric field. In the present experiment, GO was successfully reduced to ErGO in the presence of 0.1 M H_3_PO_4_ at electrolysis potential of −1.2 V (vs SCE). The reduction current response appeared in the region of −0.95 V to −1.1 V corresponds to the electrochemical reduction of GO to rGO (Figure S3). Therefore, the electrolysis potential of −1.2 V was applied to ensure the effective reduction of GO to rGO. At this potential, however, gas evolution will also take place, because hydrogen evolution from the copper surface occurs at around −0.4 V to −0.6 V (vs SCE) in acidic condition^40,41^. In the present study, simultaneous electroreduction of GO and gas evolution occurs, leading to the generation of porosity within the deposited ErGO sheets (as shown in Fig. 1). The partial overlapping of flexible graphene sheets resulted in the formation of physical cross-linking sites of the pErGO.

### Physicochemical properties of pErGO@Cuf materials

**Figure 2:**
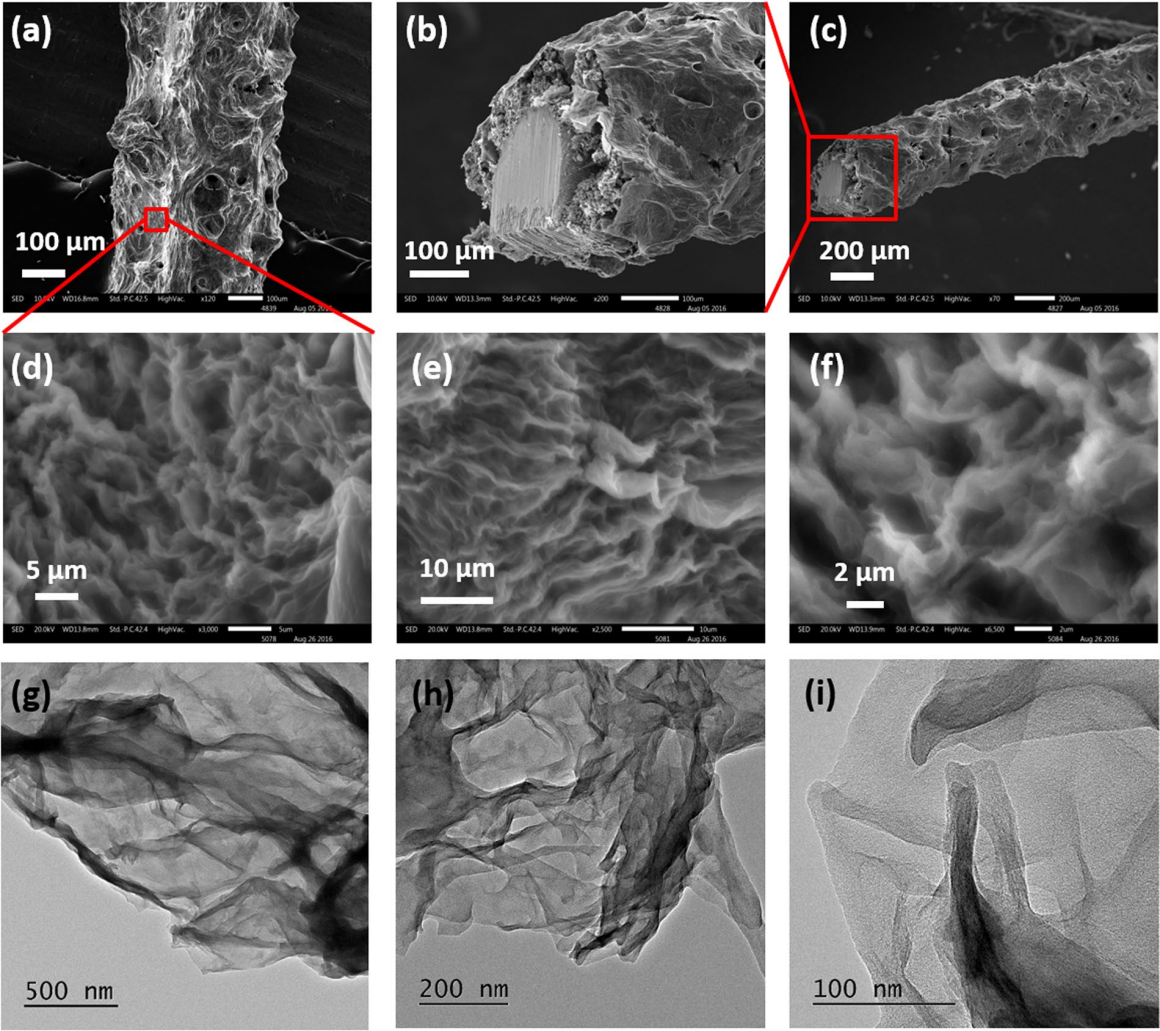
SEM images of the pErGO@Cuf/Cu-wire electrode captured at different magnifications; (**a**) shows vertical view, (**b**,**c**) show cross-sectional view of the pErGO-based wire electrode. (**d**,**e**) and (**f**) show magnified view of image (**a**) at higher resolutions highlighting vertically grown interconnected graphene sheets developed on Cuf. TEM images (**g**,**h**) and (**i**) of pErGO at different magnifications.

The microstructures and morphology of the porous graphene along with Cuf were studied with scanning electron microscopy (SEM). SEM micrographs were acquired in different magnifications to analyze the surface morphology of the as-developed Cuf (Figure S4, supplementary information) and pErGO sheets (Fig. 2) on the Cuf modified Cu wire. The SEM images of Cuf show symmetrical distributions of pores with an average size of 40 µm (Figure S5a, supplementary information). Optimum electrolysis conditions lead to the deposition of porous three-dimensional rGO sheets on the Cuf template. As can be observed from the higher magnifications of SEM images, the graphene sheets are interconnected to each other (Fig. 2d–f). In some region of the networks, the sheets are vertically oriented (Fig. 2e). The transmission electron microscopic (TEM) measurement was carried out to investigate the three-dimensional connectivity of the graphene sheets further. Figure 2(g–i) shows graphene sheets with wrinkles, and they are connected to each other. The rim, highly wrinkled and overlapped graphene sheets seemingly occur upon the growth of aqueous droplets and evolution of gas under constant potential. Electrochemically deposited rGO have the advantage of not suffering from irreversible agglomeration, unlike chemically modified graphene. They directly attach to the electrode surface after reduction for being insoluble in the immediate GO solution^42^. Additionally as reported in our previous work^23^, the Cuf template itself provides a novel platform for the deposition of ErGO at the boundary, inside the pores and on the wall of the Cuf thus introducing the much-needed porosity for EDL charge-storage^23^. Average pore diameter of vertically grown pErGO sheets was estimated as 2.5 µm (Figure S5b, supporting information). These micrometer-sized pores allow complete access to the electrolyte ions in order to store charge by sheer physical adsorption.

**Figure 3:**
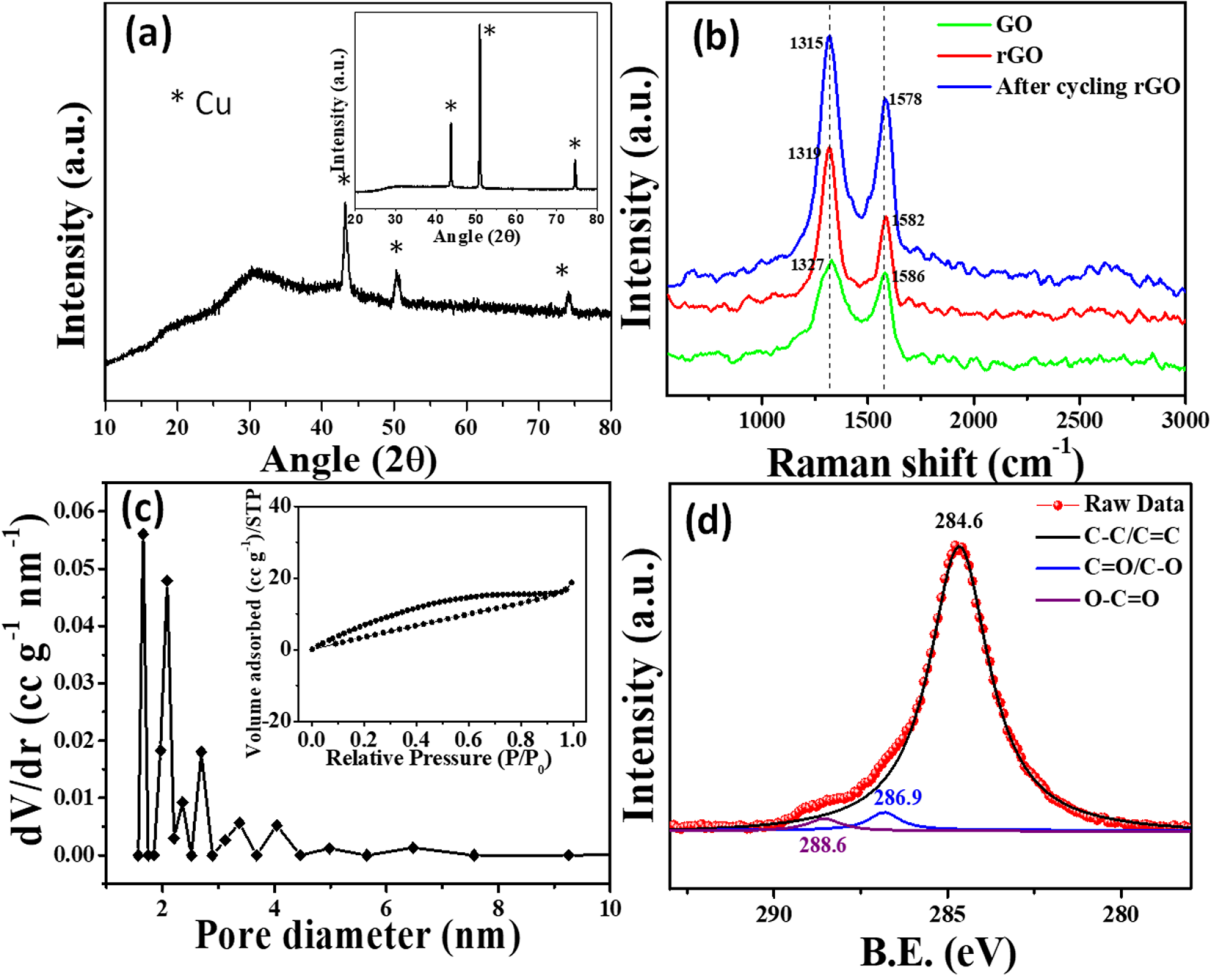
(**a**) XRD spectra of pErGO on the substrate (Cuf/Cu-wire) Inset shows the XRD pattern of Cuf/Cu-wire. (**b**) Raman spectra of GO (green), pErGO before (red) and after (blue) electrochemical cycling. (**c**) BJH pore size distribution plot of pErGO showing mesopores. (**d**) C1s core level spectra of the pErGO material.

The structural analysis of the pErGO material deposited on the Cuf/Cu wire was performed by X-ray diffraction (XRD) and Raman spectroscopy. The XRD spectra (using Cu-K_α_ = 1.542 Å) of the active material (pErGO) on the substrate (Cuf/Cu-wire) exhibits characteristic diffraction peak at 2θ = 30.2° corresponding to the (002) plane of rGO. Other peaks correspond to the substrate’s (Cu) crystal planes (shown inset of Fig. 3a). A sharp characteristic peak at 2θ = 43.5° corresponds to (111) plane, 2θ = 51° corresponds to (200) plane while the peak at 2θ = 74.5° corresponds to the (220) plane of Cu (Fig. 3a, inset).

Raman spectral analysis was carried out to investigate the structural transformation of GO during different stages of electrochemical reduction to rGO (Fig. 3b). Conjugated carbon-carbon double bonds in carbon structures lead to high Raman intensities. Though for pure graphite crystal, first-order Raman scattering mode, D-band is not active; commercially available graphitic flakes show D-band at 1333 cm^−1^ because of edge defects^43^. The D peak indicates a decrease in symmetry due to the defects on the edges. G-band comes at 1580 cm^−1^ corresponding to the degenerate in- plane E_2g_ optical mode at the center of the Brillouin zone of graphitic carbon^43^. The D- and G-bands of GO (green) were observed at 1327 and 1586 cm^−1^, respectively (Fig. 3b). This can be attributed to the introduction of defects caused by functional groups (D-band) and the presence of isolated double bonds (G-band). The D- and G-bands were red-shifted to 1319 and 1582 cm^−1^ (red curve, Fig. 3b), respectively, after electrolysis; which indicates the electrochemical reduction of GO to rGO. After electrochemical cycling, the D- and G-bands were further red-shifted to 1315 and 1578 cm^−1^ (blue curve, Fig. 3b). The intensity ratio of D to G bands, *I*_D_/*I*_G_ value was 1.12 for GO, while that of pErGO was increased to 1.53. In the case of pErGO material after electrochemical cycling, *I*_D_/*I*_G_ value was further increased to 1.85. The red-shifting of G-band and increased *I*_D_/*I*_G_ value confirm effective electrochemical reduction of GO and the restoration of isolated sp^2^ domain in the graphitic structures of the pErGO material^44,45^.

The specific surface area of the pErGO material was measured by Brunauer–Emmett–Teller (BET) adsorption-desorption isotherm and found to be 51 m^2^ g^−1^. N_2_ adsorption-desorption analysis is presented in the inset of Fig. 3c. A type-III isotherm is to be observed with an almost H3-type hystersis loop (inset of Fig. 3c) over a wide relative pressure range, being characteristic of mixed pore systems^46^. This type of loop appears usually because of non-rigid aggregates or plate-like particles forming slit-shaped pores, including pores in the micropore region^47^. BJH (Barrett, Joyner & Halenda) pore size distribution of the active material is to be observed in Fig. 3c. Average pore size was determined to be around 1.7 nm with a pore volume of 0.034 cc g^−1^.

X-ray photoelectron spectroscopy (XPS) was performed to measure the elemental composition of the as developed pErGO material. The C1s core-level spectra of X-ray photoelectron spectroscopy were used for the chemical analysis of the electrochemically reduced graphene oxide. The deconvoluted spectra is presented in Fig. 3d, which shows three peaks centered at 284.6, 286.9 and 288.6 eV corresponding to the C=C/C-C bonds (of the sp^2^ carbon in the basal plane of rGO), C-O/C=O and O-C=O functionalities, respectively^48,49^. As can be observed in the Fig. 3d, there is a significant decrease in the intensity level of C-O components with respect to the C-C bond intensity which clearly indicate efficient electrochemical reduction of GO to rGO^50^.

**Figure 4:**
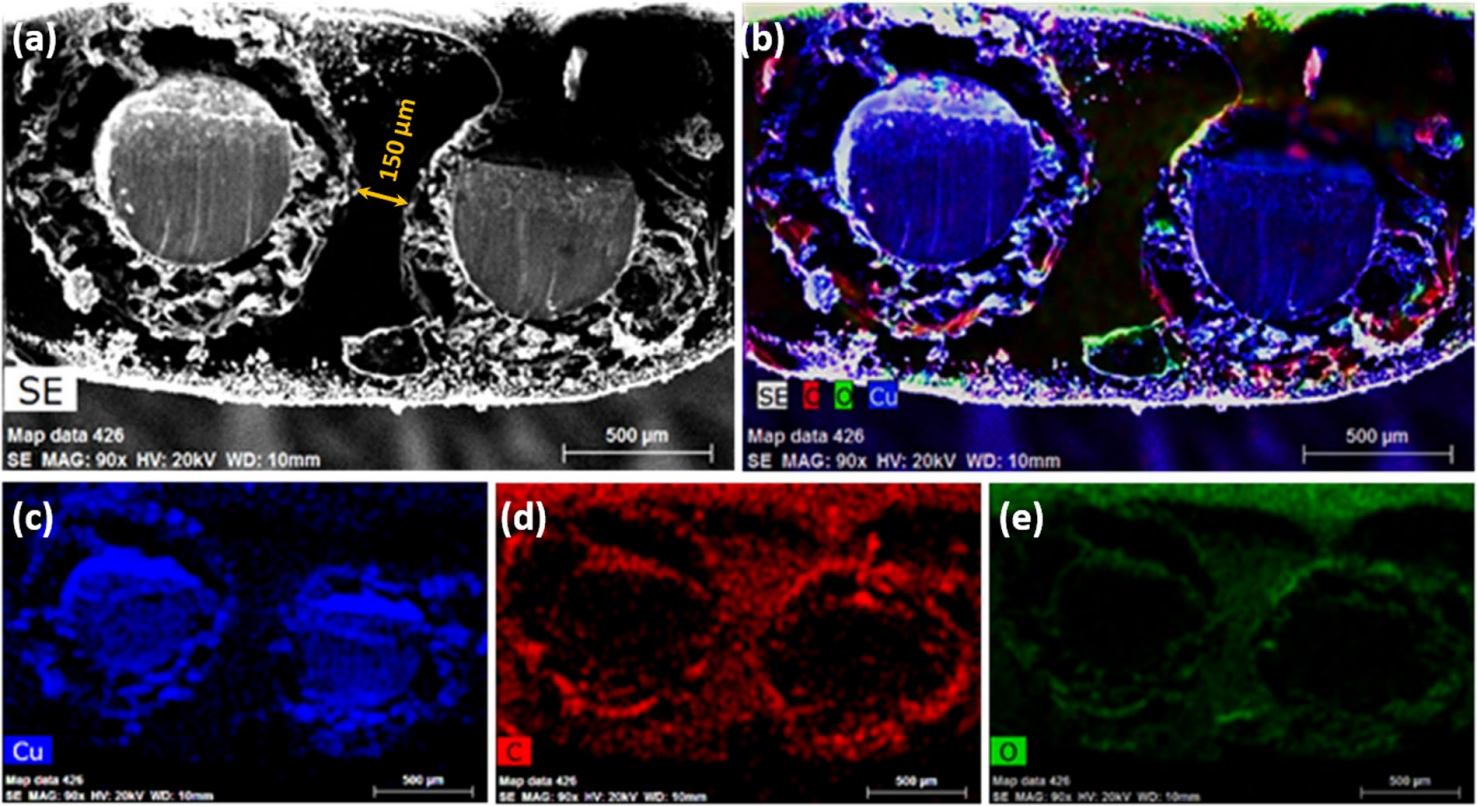
Elemental mapping of the intertwined pErGO@Cuf/Cu-based supercapacitor device. (**a**) Cross-section and (**b**–**e**) elemental mapping showing the presence of Cu, C & O elements in the fabricated device.

The intertwined solid-state assembly of pErGO electrodes covered with PVA/H_3_PO_4_ gel electrolyte was subjected to cross-sectional SEM analysis and elemental mapping. Fig. 4a shows the cross-sectional SEM image of the solid-state device, where both electrodes are well separated by the gel electrolyte, which circumvents any possible electrical shorting. The gap between two electrodes that is the thickness of the electrolyte layer was measured as 150 µm (Fig. 4a). The element distribution, i.e. distribution of copper, carbon, and oxygen was carried out by EDX mapping of the cross-section of the solid-state device (Fig. 4b–e). As can be seen from Fig. 4c–e, the core of a single electrode is composed of copper, and the shell of the electrode is mainly composed of carbon which confirms the presence of pErGO material on Cuf/Cu wire. The minute occurrence of oxygen is probably due to PVA/H_3_PO_4_.

### Electrochemical behavior of pErGO@Cuf/Cu-wire

**Figure 5:**
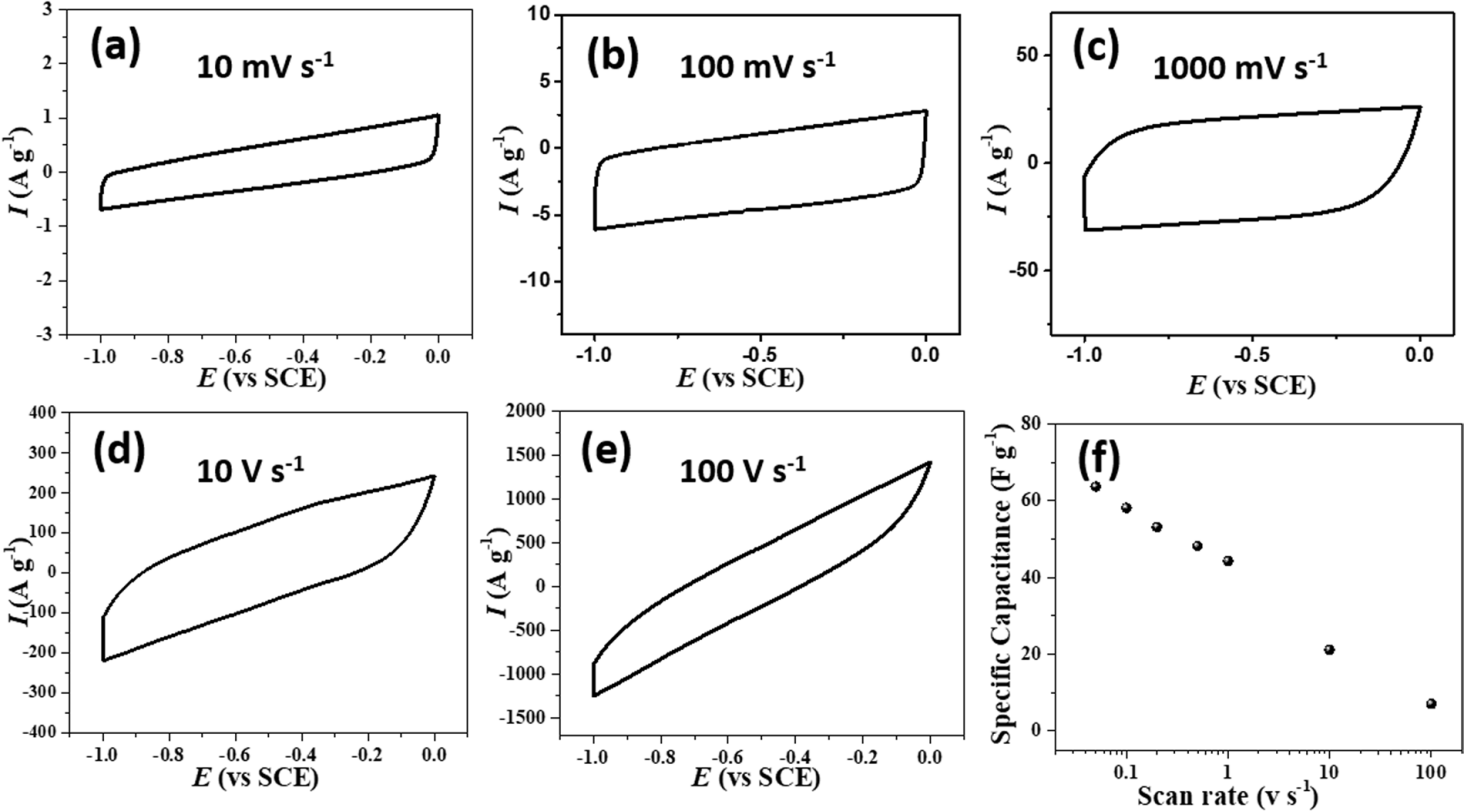
(**a**–**e**) Typical cyclic voltammetric response of the pErGO-based solid-state device at different scan rates starting from 0.01 V s^−1^ to 100 V s^−1^. (**f**) The dependence of specific capacitance on scan rate is shown with a semi log plot.

To explore the capacitive response of the fabricated wire-shaped all-solid-state supercapacitor device, two symmetrical electrodes in a parallel configuration was assembled and cyclic voltammograms (CV) were recorded. CV at various scan rates, starting from 0.01 V s^−1^ to 100 V s^−1^ was performed (Fig. 5a–f and figure S6, supporting information) to evaluate the power capability of the wire-shaped device using PVA/H_3_PO_4_ as the electrolyte in a potential window of 1 V. The wire-shaped supercapacitor displays rectangular-type CV curves indicating EDLC behavior with the proper charge propagation within the potential window −1.0 V to 0.0 V of the device. The square shape pattern of the voltammogram was maintained up to 1 V s^−1^ indicating the high instantaneous power of the device. Interestingly, the absence of any pseudocapacitive signal in CV response confirms the lack of interference from copper within the current potential window. The current response increased accordingly with an increase in scan rate (Figure S6). Fig. 5f displays the specific capacitance as a function of scan rate. The specific capacitance decreased from 76.5 F g^−1^ (at 0.01 V s^−1^) to 21.1 F g^−1^ (at 10 V s^−1^) and 7.0 F g^−1^ at a high scan rate of 100 V s^−1^, indicating an excellent rate performance of the device^51^. However, at high scan rate the rounding off of the voltammogram edges and drops in the specific capacitance was observed. Wang and Pilon recently reported a theoretical study, where they mentioned that EDL capacitance attained from CV measurements is independent of scan rate at low scan rate region^52^. However at high scan rate, i.e., diffusion-limited regime, EDL capacitance drops sharply with increasing scan rate, results a logarithmic decay of the specific capacitance as observed in Fig. 5f. Several factors including an increase of the active material resistance, active material-electrolyte interphase resistance and/or long ion-diffusion length are responsible for the sharp drop of specific capacitance at high scan rate^53,54^. The contribution of long ion diffusion length along the pErGO networks is likely the reason for low specific capacitance at high scan rate. The CV response of bare Cu wire and Cuf was also evaluated to check the possible interference of the capacitance to the pErGO materials. Interestingly it was observed that the pErGO@Cuf electrodes have 20 times higher gravimetric capacitance than Cuf and 30 times greater than Cu wire electrode (Figure S7, Supporting information). We should mention here that electrochemical cycling has a significant role in the CV pattern as well as the specific capacitance of the device (Figure S7, supplementary information). The unreduced functional group present in the reduced graphene oxides was significantly reduced after the electrochemical cycling, which is in the line with Raman data.

**Figure 6:**
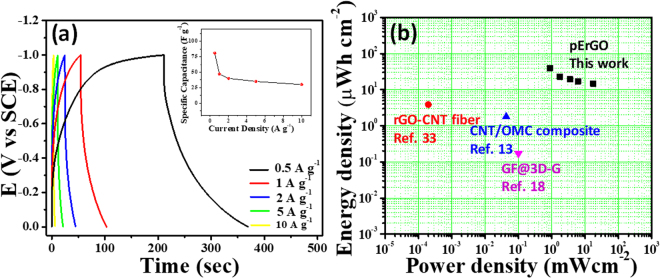
(**a**) Galvanostatic Charge-discharge recorded at different current densities from 0.5 A g^−1^ to 10 A g^−1^. Inset is the effect of increasing the current density on the specific capacitance of the device. (**b**) Ragone plot for the energy density and power density of the fabricated device compared with reported literatures^13,18,34^ on graphene-based wire/fiber supercapacitors.

Galvanostatic charge-discharge (GCD) was further studied to assess the advantage of the developed solid-state supercapacitor more precisely. Fig. 6a shows the discharge curves of the assembled device at various current densities from 0.5 A g^−1^ to 10 A g^−1^. Retention of shape of the discharge curves with little potential drop even at higher current densities is indicative of good EDL capacitor. An electrochemical window up to 1 V without alteration in the charging process is established with this flexible device. A reasonably high gravimetric capacitance of 81 F g^−1^ was achieved at a current density of 0.5 A g^−1^. The plot of specific capacitance with current densities (Fig. 6a (inset)) demonstrates that even at higher current density the specific capacitance does not drop much indicating a good rate capacity of the device. Aiming for high energy density for the developed solid-state supercapacitor was another aspect of our finding, which is described in the Ragone plot (Fig. 6b). An almost linear curve indicates achievement of sufficiently high energy density for high power densities, inherent of supercapacitors. The highest energy density of 11.25 W h kg^−1^ (equivalent to 5.6 µW h cm^−1^ and 39.3 µW h cm^−2^) was attained at a power density of 0.25 kW kg^−1^; while the highest power density of 5 kW kg^−1^ (equivalent to 2.5 mW cm^−1^ and 17.6 mW cm^−2^) was gained against a reasonably good energy density of 4.2 W h kg^−1^. The high energy and power densities of the pErGO-based flexible device are owing to the 1 V wide potential window, high ion permeability (i.e. ionic and electronic pathways through the porous materials) and conducting nature of the porous rGO together with low barrier resistance of the active material to the current collector.

The fabricated device performed better than most of the reported carbon based WSCs or FSCs; not only regarding specific capacitance but also in achieving sufficiently high energy density. It showed highest mass-specific capacitance of 81 F g^−1^ (equivalent area specific capacitance of 283.5 mF cm^−2^ and length-specific capacitance of 40.5 mF cm^−1^) at a current density of 0.5 A g^−1^; much higher than any of the reported wire/fibre based EDLCs with activated carbon^55^, graphene^18^ and CNTs^56^ as the active electrode material till date (Table 1). The length-specific capacitance of the pErGO-based wire (4 cm) supercapacitor is as high as 40.5 mF/cm, which is almost 3500 times higher than that of the ErGO on Au wire (0.011 mF cm^−1^)^24^, 2000 times that of the graphene fibers (0.02 mF cm^−1^)^18^, and even 1600 times that of CNT fibers (0.024 mF cm^−1^)^56^.

**Table 1:** Table for comparison of all carbon-based wire/fiber-shaped supercapacitors with the present work.

Materials	Electrolyte	Specific capacitance	Energy density	Power density	Working voltage	Reference
rGO/CNT core-sheath fibres	PVA/H_3_PO_4_	5.3 mF cm^−1^177 mF cm^−2^	3.84 μW h cm^−2^3.5 mW h cm^−3^	0.02 μW cm^−2^	0.8 V	^34^
3D graphene-RACNT	PVA/H_2_SO_4_	158 F cm^−3^89.4 mF cm^−2^23.9 mF cm^−1^	—	—	0.8 V	^57^
MWCNT fibres	PVA/H_3_PO_4_	13.31 F g^−1^0.015 mF cm^−1^3.01 mF cm^−2^	174.40 mA h g^−1^94.37 mA h cm^−3^	—	1 V	^15^
CNT/OMC composite	PVA/H_3_PO_4_	1.91 mF cm^−1^39.7 mF cm^−2^	1.77 μW h cm^−2^0.085 μW h cm^−1^	0.043 μW cm^−2^	1 V	^13^
ErGO on Au wire	PVA/H_3_PO_4_	11.4 μF cm^−1^0.726 mF cm^−2^	—	—	1 V	^24^
Carbon microfibre/SWCNT/ N-doped rGO	PVA/H_3_PO_4_	116.3 mF cm^−2^300 F cm^−3^	6.3 μW h cm^−3^	1.085 μW cm^−3^	1 V	^4^
GF@3D-G	PVA/H_3_PO_4_	40 F g^−1^20 μF cm^−1^1.7 mF cm^−2^	0.17 μW h cm^−2^	100 μW cm^−2^	0.8 V	^18^
AC fibre from GO	PVA/H_3_PO_4_	43.8 F g^−1^27.6 F cm^−3^	2.5 mW h cm^−3^3.96 mW h g^−1^	5 mW cm^−3^	0.8 V	^55^
Hydrothermally reduced GO on SSW	PVA/H_3_PO_4_- Na_2_MoO_4_	18.75 mF cm^−1^38.2 mF cm^−2^	2.6 μW h cm^−1^5.3 μW h cm^−2^	—	1 V	^30^
Self-assembled GA on Cu wire	PVP/NaI	62.3 F g^−1^12.5 mF cm^−1^	—	—	1 V	^33^
**pErGO@Cuf/Cu wire**	**PVA/H**_**3**_ **PO**_**4**_	**81 F g** ^**−1**^ **40.5 mF cm** ^**−1**^ **283.5 mF cm** ^**−2**^	**11.25 W h Kg** ^**−1**^ **5.6 μW h cm** ^**−1**^ **39.3 μW h cm** ^**−2**^	**5 kW Kg** ^**−1**^ **2.5 mW cm** ^**−1**^ **17.6 mW cm** ^**−2**^	**1 V**	**This work**

GO: graphene oxide; rGO: reduced GO; ErGO: electrochemically rGO; MWCNT: multi-walled carbon nanotube; SWCNT: single walled carbon nanotube; RACNT: radially aligned CNT; OMC: ordered mesoporous carbon; GF: graphene fiber; AC: activated carbon; SSW: stainless steel wire; GA: graphene aerogel; PVP: polyvinylpyrrolidone; PVA: poly-vinyl alcohol.

We should mention here that our pErGO-based wire supercapacitor even shows higher specific capacitance than that of hybrid carbonaceous materials based wire/fiber supercapacitor^4,29,32,55,57^ reported in recent years and it is still among the best. While talking about the most sought-after feature of an SC, i.e.; energy density; the pErGO-based device performed remarkably well (39.3 µW h cm^−2^) than rGO/CNT core-sheath fibers (3.84 µW h cm^−2^)^34^, CNT/OMC composite fibers (1.77 µW h cm^−2^)^31^ and graphene fiber (GF) on 3D graphene (0.17 µW h cm^−2^)^18^ (as shown in Fig. 6b) without affecting its inherent properties like power-density (17.6 mW cm^−2^) and long cycle-life. The pErGO-based flexible supercapacitor achieved a better mass-specific capacitance of 81 F g^−1^ than the recently reported self-assembled graphene aerogel on Cu-wire (62.3 F g^−1^)^33^. It is interesting to note that our WSC system is better than other WSCs/FSCs, where similar deposition method like electrochemically reduced GO on Au-wire^24^ (C_sp_ 0.726 mF cm^−2^) or GF@3D-graphene^18^ (C_sp_ 1.7 mF cm^−2^) have been reported.

**Figure 7:**
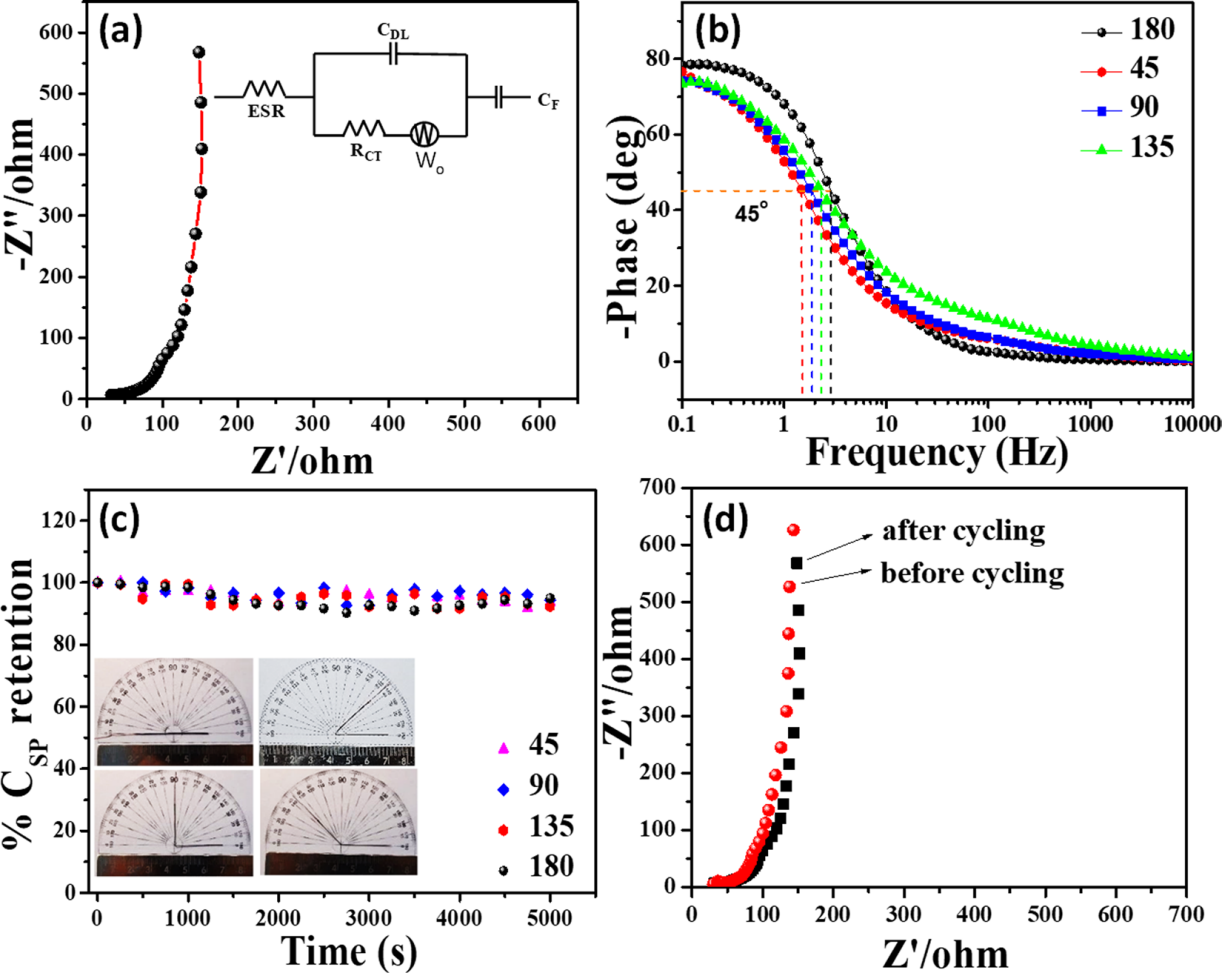
(**a**,**b**) EIS analysis of the fabricated supercapacitor device; (**a**) shows Nyquist plot recorded in the frequency range of 100 kHz to 0.01 Hz and (**b**) shows the Bode plot of the device at different bending angles. (**c**) Durability test of the device recorded in the form of % retention of capacitance with the number of cycles. Inset shows the optical image of the device at different bending angle and corresponding stability data are given. (**d**) Nyquist plot before and after 5000 cycles.

Electrochemical impedance spectra (EIS) analysis was performed to understand the kinetic feature of the ion diffusion responsible for charge storage property of the electrode. The Nyquist plot of pErGO electrode is demonstrated in Fig. 7a. The plot does not show any semicircular region, indicating the material possess low faradaic resistances in the electrolyte^44^. The Nyquist plot in Fig. 7a initially intersects the real axis at 45^ο^, it may be attributed to the Warburg impedance, which arises in a porous electrode when accessed by an electrolyte ion^58^, as evident from the SEM images (Fig. 2d–f). At lower frequency region, a vertical curve indicates a simple EDLC system. The Nyquist plot was fitted well with an equivalent circuit as presented in the inset of Fig. 7a by the following equation^59^.

$$Z={R}_{s}+\frac{1}{{j}_{\omega }{C}_{DL}+\frac{1}{{R}_{CT}+{W}_{0}}}-j\frac{1}{\omega {C}_{F}}$$

The fitted data are provided in a tabular form in Table S1. ESR is the equivalent series resistance comprising resistance of the electrode materials, electrolytes, current collectors and contact resistance; R_ct_ is the charge transfer resistance, C_DL_ is the double layer capacitance, C_F_ is the faradaic capacitance and W_o_ is the finite-length Warburg diffusion element, which is expressed as, A/(jω)^n^, where A is the Warburg coefficient, ω is the angular frequency and n is an exponent^59^. Fig. 7b is the Bode plot, where phase shift is 90° in the low-frequency region indicating the double layer charge storage property and almost absence of faradic type charge storage phenomena of the materials^60^. The dependence of phase angle on the frequency was evaluated and is presented in Fig. 7b. The characteristic frequency at which the phase angle reaches 45° is known as the knee frequency (*f*_0_), where the capacitive and the resistive impedances are equal^61^. After this point, at higher frequencies, the supercapacitors show more resistive behavior. The relaxation time (*τ*_0_ = *f*_0_^*−1*^) indicates the minimum time required to discharge all the energy from the device with an efficiency of greater than 50%^62^. The relaxation time of the device was found to be 348, 435, 533, and 664 ms at a bending angle of 180°, 135°, 90°, and 45°, respectively (Table S2). It is well known that higher the knee frequency, higher is the rate capability (lower is the relaxation time)^61^. As can be observed in Fig. 7b, although the relaxation time increases with increasing strain in the pErGO electrodes, the change is very minimal, indicating the integrity of the device is maintained even at bending states. Moreover, the relaxation time for the dropcast rGO material was found to be 2375 ms, much higher (~7 times) than our pErGO active electrode material (348 ms), as presented in Figure S8. It is thus evident from the Bode plot (Figure S8) that pErGO has got faster frequency response capabilities, with shorter ionic transport paths that lead to reduced internal resistance and higher electrical conductivity^62,63^. The rate capability of the electrochemically prepared pErGO material is thus significantly higher than the chemically prepared rGO material, which demonstrates the benefit of simultaneous electrochemical reduction and deposition method over simple dropcasting method.

The most important part of the present study is the stability of the fabricated flexible supercapacitor, studied up to 5,000 GCD cycling (Fig. 7c) at a fixed current density of 5 A g^−1^. Retention of capacitance was plotted against number of cycles (Fig. 7c) and revealed that 94.5% of initial capacitance retained after 5000 cycles. High retention of capacitance can be attributed to the maintenance of structural integrity of the pErGO after continuous GCD cycling as evident from the SEM images (Figure S9, supplementary information). The electrochemical behavior of the device after 5000 cycling was also checked with CV and impedance analysis (Figure S10, supplementary information and Fig. 7d respectively). A little decrease in the area under the curve after cycling was observed (Figure S10, supplementary information). No significant changes in the impedance curve (Fig. 7d) were observed after continuous cycling indicating excellent stability of the device. The results demonstrate the high reliability of the fabrication method as well as the stability of the pErGO materials on Cuf/Cu wire electrode. To further investigate the stability of the device, GCD cycling (5000 cycles) was studied under mechanical bending at different bending angle as shown in inset of Fig. 7c. Interestingly, it was observed that approximately 92–93% specific capacitance retained (Table S3) after 5000 cycles even at different bending angle. This impressive retention of the specific capacitance at different angle is promising for various practical application of the wire-shaped supercapacitor device.

**Figure 8:**
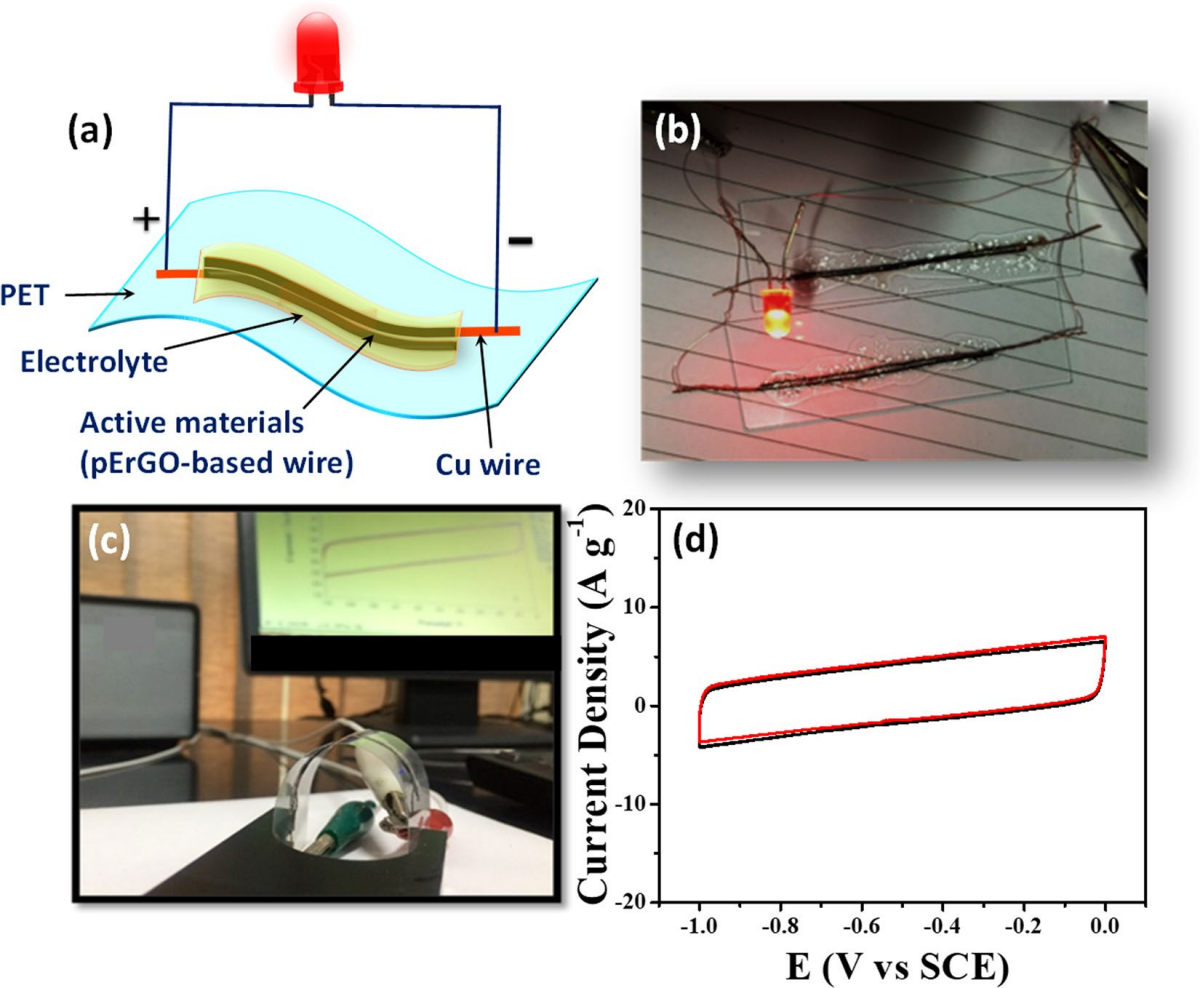
(**a**) Schematic representation of the fabricated all-solid-state symmetrical supercapacitor device on a flexible PET sheet. (**b**) Digital photograph of the two devices connected in parallel, which is powering the commercially available red LED. (**c**) Digital picture showing the flexibility of the fabricated device during cyclic voltammetry measurements. (**d**) Cyclic voltammetric responses of the device in flat mode (black) and in bent mode (red).

The ideal way to check the performance of an efficient supercapacitor device is that first it can be charged and then discharge to operate various commercial electronic devices. Two pErGO-based solid-state device (each length 4 cm) were assembled on a PET sheet and connected in-parallel as shown in Fig. 8a,b. The final device is then first connected with a 3 V battery for charging for 30 s. After the device was fully charged, the supercapacitor device was able to power a commercially available red LED.

### Flexibility and scalability of the device

**Figure 9:**
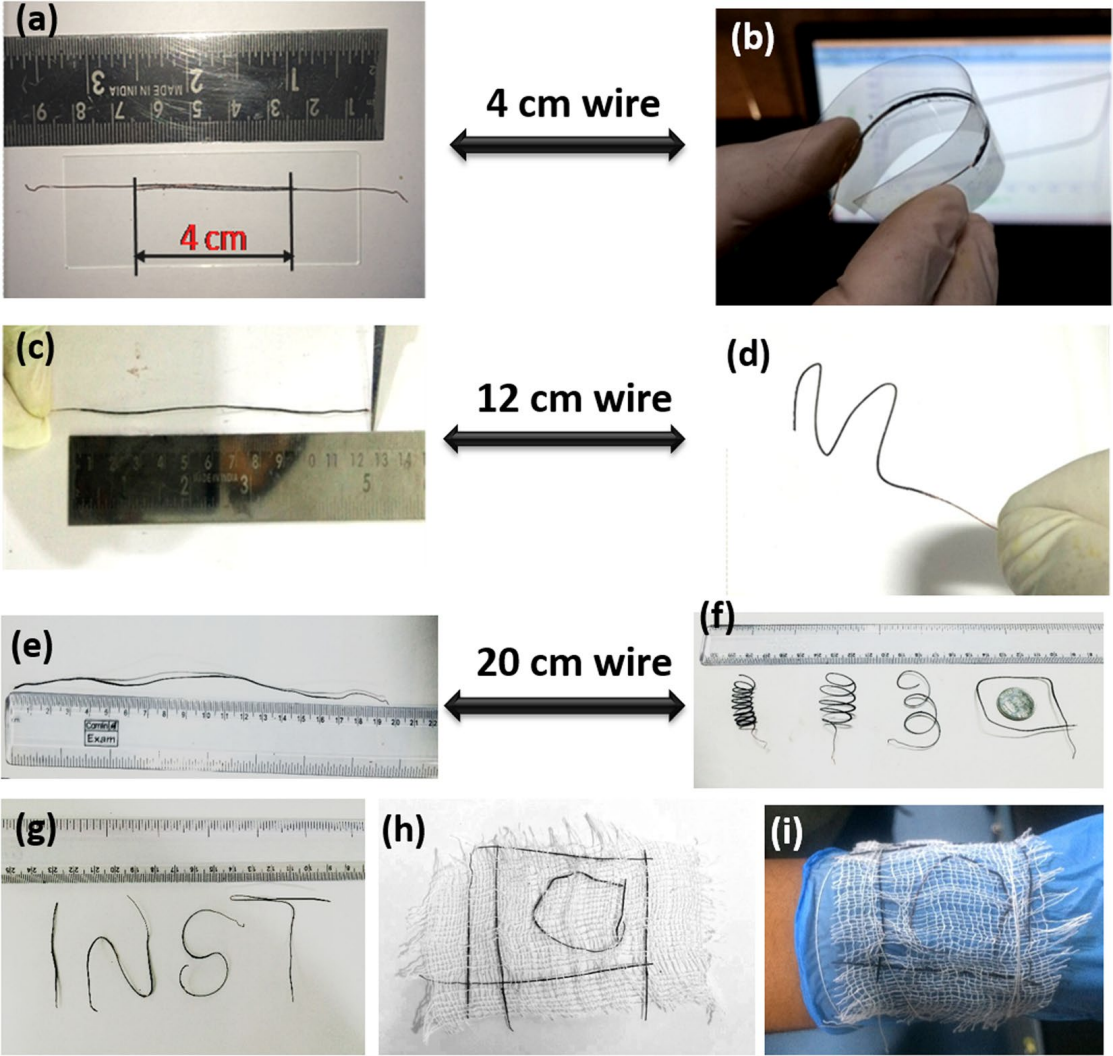
Flexibility and scalability of the device: A 4 cm modified Cu-wire as used in the original device fabrication (**a**,**b**); which can be scaled up to 12 cm (**c**,**d**) and 20 cm (**e**,**f**). The wires can be shaped into various geometries as in (**d**,**f** and **g**). Modified wires (12 cm) woven into a piece of fabric for its possible application in textile/wearable devices (**h**,**i**).

The integration of co-axial, highly flexible and scalable device has potential applications in biomedical devices, antimicrobial textiles, garments and wearable electronics^10,64^. To test the flexibility and bendability of pErGO-based device, CV measurements at 100 mV s^−1^ were carried out for the straight (planar) and bent states. Figure 8c shows the digital photograph while taking the CV at bent mode. The overlapping CV corresponding to the straight and bent modes at 100 mV s^−1^ is defined in Fig. 8d. The area under the CV curves from the straight and bent devices are almost identical, obviously suggesting that the pErGO-based supercapacitor coated with the polymer gel electrolyte is highly flexible in nature. To check the scalability of the device without sacrificing its flexibility, pErGO was modified on 12 cm and 20 cm long Cuf/Cu wire electrode. The pErGO modified long wires were subjected to different shapes and geometries (zig-zag, spiral, and alphabets) as shown in Fig. 9b,d,f and g. These consequences of flexibility and scalability reflect that the pErGO-based wire is completely flexible and wearable. This performance can be accredited to the high mechanical flexibility of the electrodes and the solid gel electrolyte. The pErGO-based all-solid-state flexible SCs were further engaged to travel their applications in flexible and wearable electronic devices that can be intertwined into textiles by a simple conventional weaving method. Fig. 9h shows that six modified wires (12 cm long) were integrated into the cotton fabric to demonstrate the proper insertion of modified device into cotton threads. As the device is flexible and wearable, the cotton fabric with weaved device is freely bent and worn on the wrist (Fig. 9i). The device is flexible, scalable and tunable to woven textile or wearable electronics; it meets the requirement of high energy and power device with weavability into textile and electronics for future applications.

## Conclusion

In summary, we have developed porous electrochemically reduced graphene oxide networks on copper wire supported copper foam by electrochemical method and used it as an active material to fabricate flexible all-solid-state supercapacitor device. The symmetrical EDLC device was designed using the two co-axial pErGO@Cuf/Cu-wire electrodes with PVA/H_3_PO_4_ supported gel electrolyte. The device was able to deliver high specific capacitance of 81 F g^−1^. This is one of the highest Csp exhibited by a solid state WSCs/FSCs known till date to the best of our knowledge. The fabricated wire-shaped supercapacitor is mechanically flexible and does not alter its electrochemical performances upon bending of the device at different angles. The whole electrode setup was replicated on a flexible substrate like PET with PVA/H_3_PO_4_ to show the flexibility performances of the device. To explore more practical applications, we powered up a LED bulb arranging two devices in parallel connection. The device is flexible and could be assembled into different geometries with higher lengths, which is suitable for textile/wearable applications. Vertically aligned open-network porous structures along with low barrier resistance of the active material to the Cu wire are responsible for the high charge storage capacity.

## Electronic supplementary material

Supplementary Information
