# Supplementary material for: High-performance flexible supercapacitors based on electrochemically tailored three-dimensional reduced graphene oxide networks
Source: Sci Rep. 2018 Jan 12;8:640. doi: 10.1038/s41598-017-18593-3 (PMC5766552; doi:10.1038/s41598-017-18593-3)
Supplement: Supplementary file 1 — Supplementary Information [file 41598_2017_18593_MOESM1_ESM.pdf]

Electronic Supplementary Information

High-performance flexible supercapacitors based on electrochemically tailored three-dimensional reduced graphene oxide networks

*Taniya Purkait, Guneet Singh, Dinesh Kumar, Mandeep Singh, Ramendra Sundar Dey**

Institute of Nano Science and Technology (INST), Mohali-160062, Punjab, India.
Email: rsdey@inst.ac.in (R. S. Dey)

1. Reagents and Instruments

Highly pure graphite flakes (<20µm), phosphorous pentoxide (P_2O_5, >98%), potassium permanganate ($KMnO_4$) and copper sulphate ($CuSO_4$) were purchased from Sigma Aldrich and were used as received. Sulphuric acid (H_2SO_4) and phosphoric acid (H_3PO_4) were purchased from Merck chemicals India. Poly (vinyl) alcohol (M.W. 89000-98000) was purchased from Alfa Aesar (Thermo Fischer Scientific Chemicals Inc.; US). Cu wires (d = 0.25 mm) were purchased from local market. Before use, all the Cu wires were repeatedly washed with dilute nitric acid (HNO_3, Merck Chemicals India), Millipore water & finally with acetone (CH_3COCH_3, Merck Chemicals India) to obtain clean & hydrophilic surfaces. All other chemicals used were at least of analytical grade. All aqueous solution was prepared using Millipore water.

Electrochemical measurements like cyclic voltammetry, galvanostatic charge-discharge, Electrochemical Impedance Spectroscopy (EIS), etc. were performed on a CHI 660E electrochemical workstation. During electrodeposition, copper foam modified copper wire was assigned as the working electrode while the counter electrode was a platinum wire; all calculations were made against saturated calomel electrode (SCE) that was selected as the reference electrode. X-ray diffraction spectroscopic (XRD analysis) study was carried out to determine the crystal structure of the synthesized GO with the help of a Bruker D8 Advances instrument using Cu-K_α (λ=1.5406 Å) radiation in the range 2θ from 5° to 80°.The surface morphology and the elemental composition of the fabricated electrode material was investigated using Scanning Electron Microscopy (SEM; JeolJSMIT300) equipped with a Bruker XFlash6130 Energy Dispersive X-ray Spectroscopy (EDS). The structural changes of the active material were studied with the

help of Raman spectral analysis on a WITEC Focus Innovations Alpha 300 Raman confocal microscope. X-Ray photoelectron (XPS) spectroscopy was performed on a K-Alpha plus XPS system by ThermoFisher Scientific instruments in an ultrahigh vacuum chamber ($7X10^{-9}$ torr) using Al K_{α} radiation (1486.6 eV). Nitrogen adsorption-desorption analysis was done at 77 K on an Autosorb iQ2 instrumental setup to examine the surface area by Brunauer Emmett Teller (BET) method. The pore size distribution was measured by the Barrett, Joyner & Halenda (BJH) method. The samples were degassed at 150 °C for more than 12 h under vacuum conditions. Transmission Electron Microscopy (TEM) studies were carried out on a JEM2100 instrument suitable for High-Resolution TEM (HRTEM) studies.

2. Synthesis of graphene oxide

Graphene-oxide was synthesized via two-step approach according to our previously developed method.[1] In first step, pre-oxidised graphite powder was obtained from graphite power followed by its subsequent oxidation and exfoliation in second step to achieve highly dispersed and well-separated graphene oxide sheets.[2] In the initial step, Graphite powder (5 g) was slowly added into concentrated H_2SO_4 solution (10 ml) containing P_2O_5 (2.5 g) and $K_2S_2O_8$ (2.5 g) kept in a hot water bath (80°C) under strong stirring for 3 h. After cooling to room temperature and diluting with Milli-Q water, a dark green mixture was obtained. The mixture was then filtered and washed several times until pH of waste solutions reaches neutral. Pre-oxidized graphite powder was collected and dried in air at room temperature overnight. In the second step, pre-oxidized graphite powder (1.0 g) was slowly added to concentrated H_2SO_4 solution (25 ml) maintaining the temperature at 0 °C. Solid $KMnO_4$ (3.0 g) was then added to the mixture under slow

stirring and maintaining the temperature below 20 °C. After removing the ice-water bath, the mixture was heated at 35 °C under stirring for 2 h, to which Milli-Q water (50 ml) was then added. After a few minutes, Milli-Q water (150 ml) and 30% H_2O_2 (5 ml) solution were further added to the mixture, leading to the solution colour changed rapidly to bright yellow. The mixture was then washed with 10% HCl solution (v/v, 250 ml) and filtered to remove residual metal ions. The raw GO suspended in Milli-Q water was centrifuged at a high rotation speed (12000 rpm). The supernatant containing highly dispersed and stable GO nanosheets was collected. To remove residual salts and acids, the sample was further dialyzed using a dialysis tube (with a cut-off molecular weight of 12000-14000) for 7 days by regularly replacing water bath with fresh Milli-Q water 2-3 times per day. The dialyzed GO solution was stored in a reagent bottle in refrigerator and was stable for 6 month with no colour change and precipitation observed.[3]

3. Calculations and formulas of all-solid-state wire supercapacitor.

The specific capacitance (C_{SP}) can be calculated from cyclic voltammetry via the equation (1)

$$C_{SP} = \frac{I_{avg}}{mv} \qquad (1)$$

where I_{avg} is the average current obtained from cathodic and anodic sweeps, υ is the scan rate, and m is the active mass of the pErGO material interdigitated onto the electrode surface.

The specific capacitance, C_D of the supercapacitor device was calculated from galvanostatic charge-discharge curves[4–7] by equation (2) as following

$$C_D = \frac{I\Delta t}{\Delta V}\frac{1}{\langle M|L|A\rangle} \qquad (2)$$

where, I (in A) is the discharge current, Δt (in s) is the discharge time, M (in g) is the active mass of device, L (in cm) is the length of the electrode or device, an (in cm^2) is the area of the device and ΔV (in V) is the working voltage.

The equation can express the energy density (E) and power density (P) of a supercapacitor device, (3) moreover, (4) as following

$$E = \frac{\Delta V^2}{2\times 3600}(C_{sp})_{\langle M|L|A\rangle} \quad (3)$$

$$P = \frac{E\times 3600}{\Delta t} \quad (4)$$

Where $(C_{sp})_M$, $(C_{sp})_L$ and $(C_{sp})_A$ is the mass, length, and area specific capacitance, respectively.

Supporting Data

Among different concentrations of GO aqueous suspension, deposition for 200 seconds at 5 mg mL^{-1} proves to be the best, yielding highest C_{sp} of the pErGO modified supercapacitor electrodes. Fig. S1 shows the effect of GO concentration on the specific capacitance. All CV experiments were carried out in 1 M phosphoric acid solution.

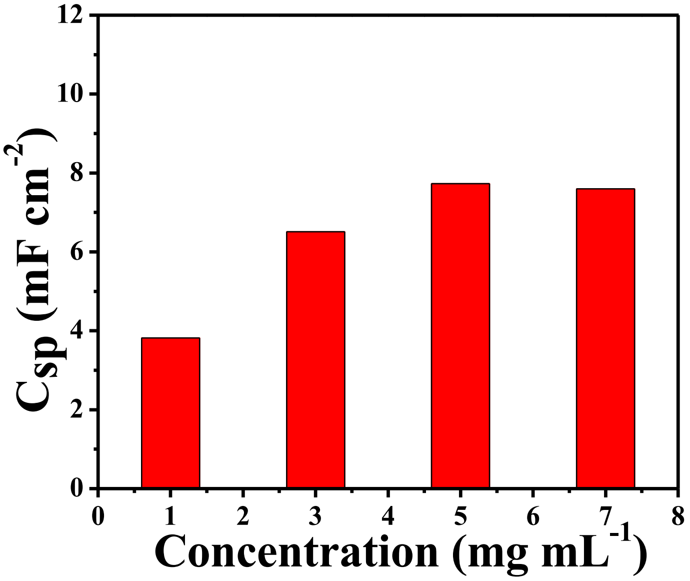

Fig. S1. Effect of concentration of GO aqueous suspension on the specific capacitance of the developed pErGO electrode material.

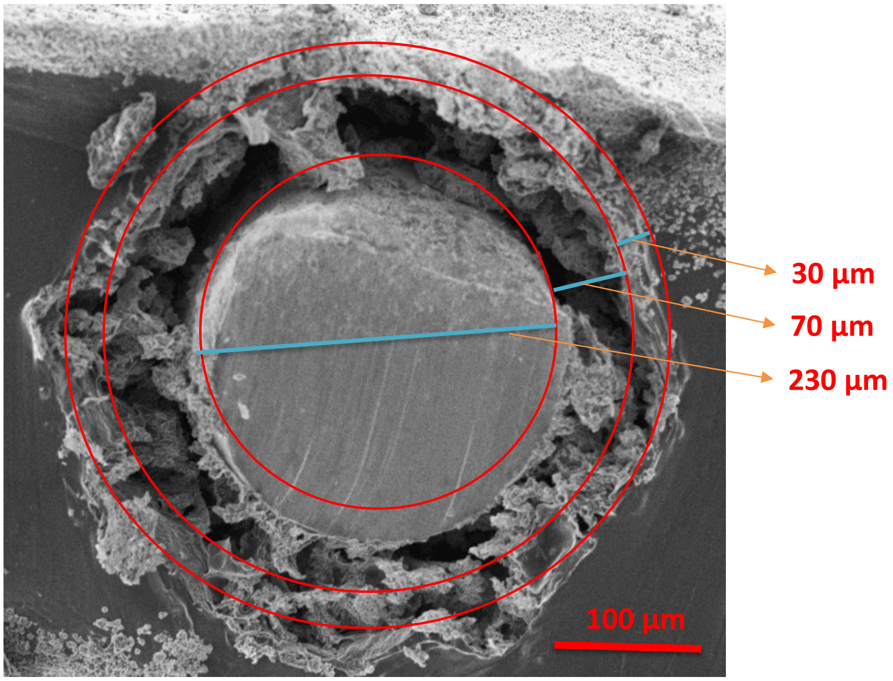

Fig. S2. Cross-section of a modified electrode clearly showing the diameter of each layer formed on Cu wire.

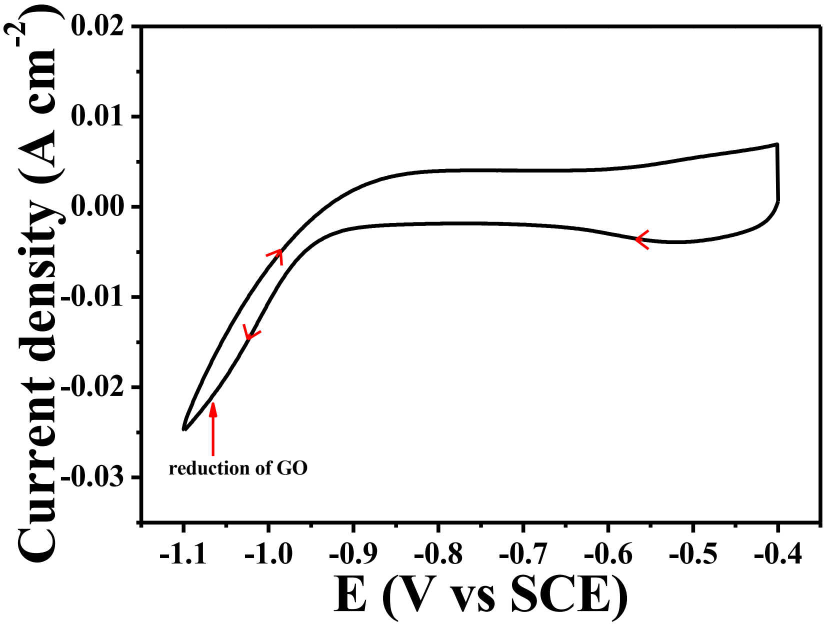

Fig. S3. Cyclic voltammogram of GO aqueous suspension in 1 M H_3PO_4.

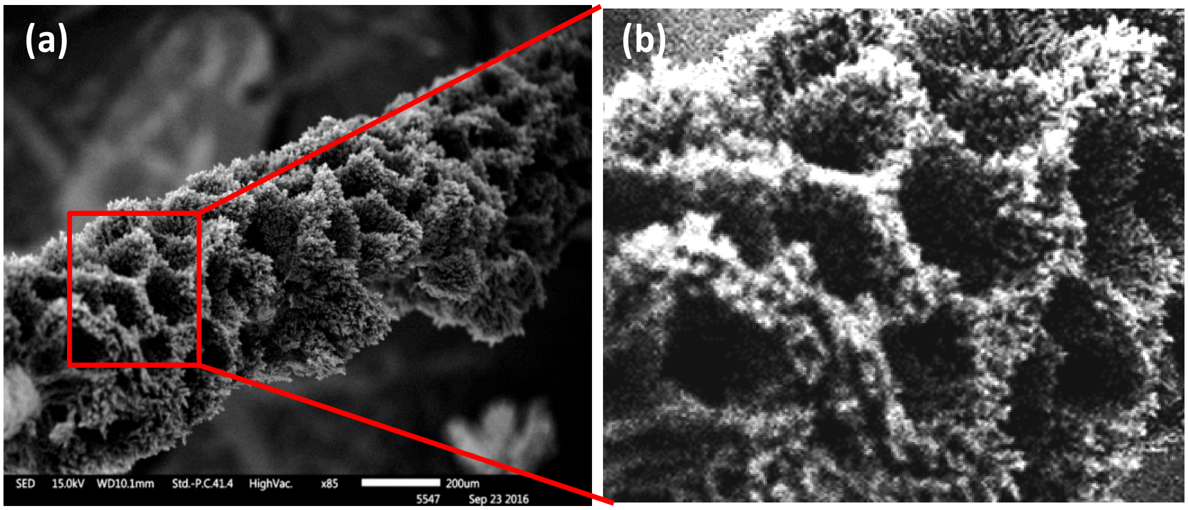

Fig. S4. SEM images captured in different magnifications of the Cuf modified Cu-wire electrode. (a) Vertical view of Cu-foam (Cuf) modified Cu-wire at 100mm magnification. (b) Magnified view of Cuf showing almost spherical pore allowing deposition of rGO in and across it.

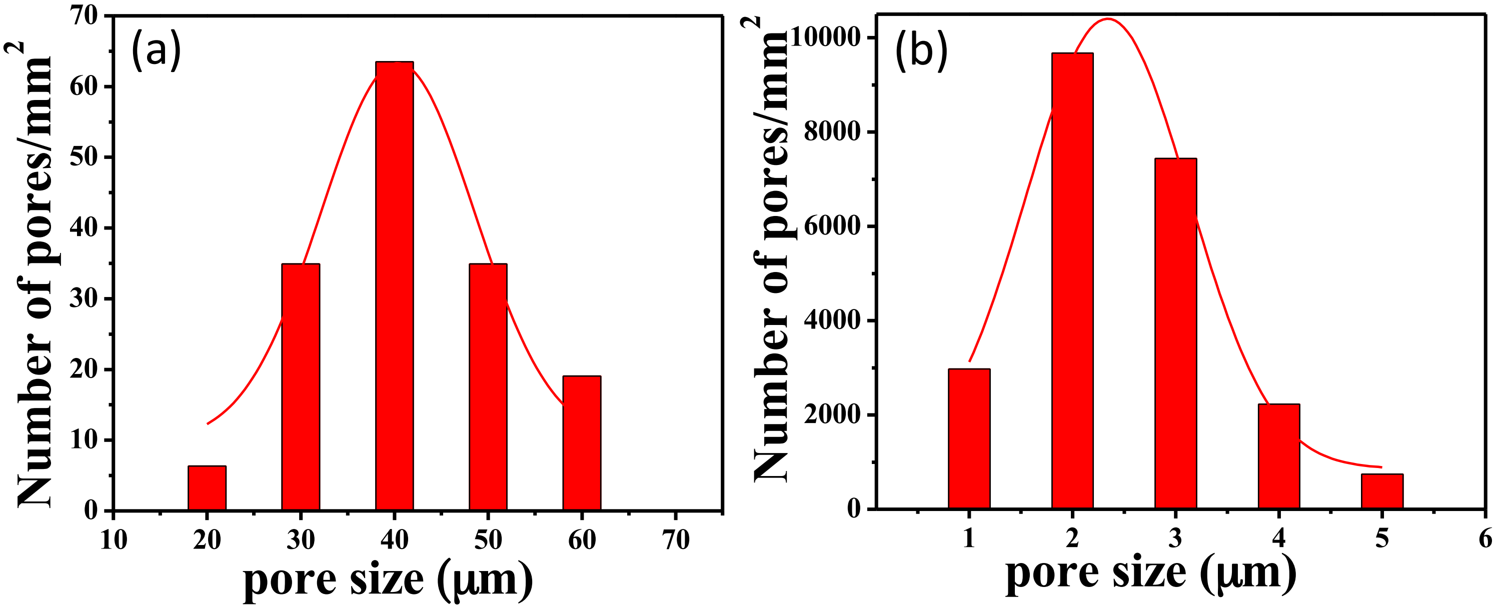

Fig. S5. Histograms of the pore size distributions of (a) Cuf, (b) pErGO nanosheets.

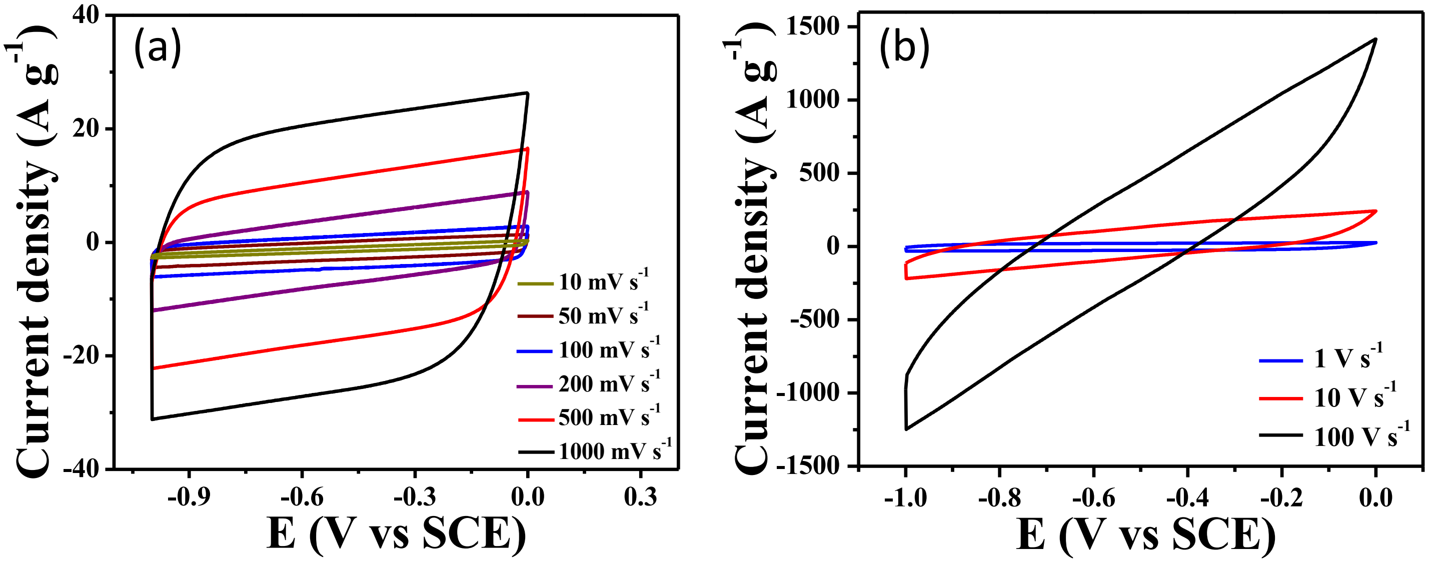

Fig. S6. CV response of the pErGO@Cuf electrode at various scan rate. (a) Scan rate was 0.01 to 1 v s^{-1} and (b) 1 to 100 v s^{-1}.

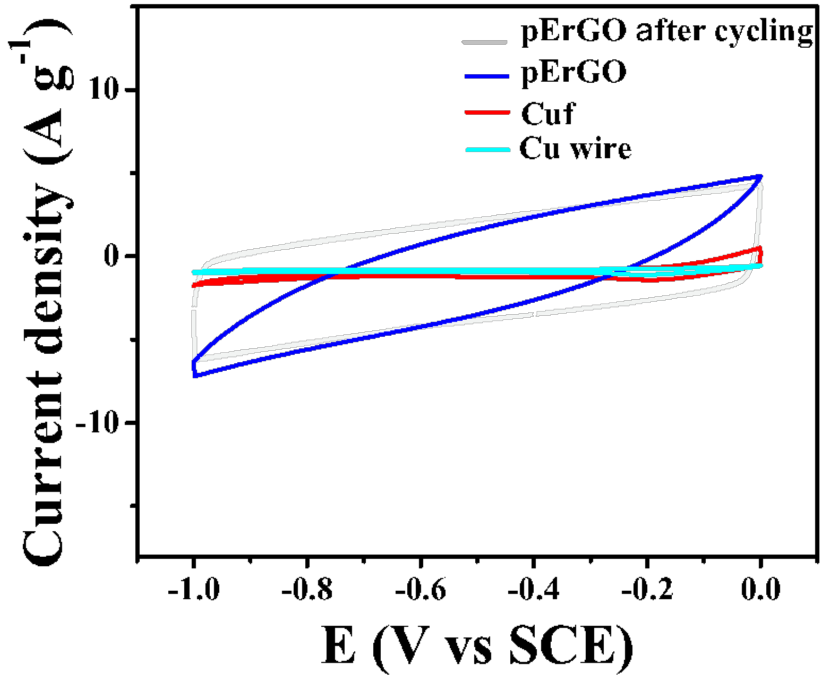

Fig. S7. CV response of the Cu wire (navy blue), Cuf (red), pErGO@Cuf (blue) and pErGO@Cuf after CV cycling (grey). Scan rate: 100 mV/s.

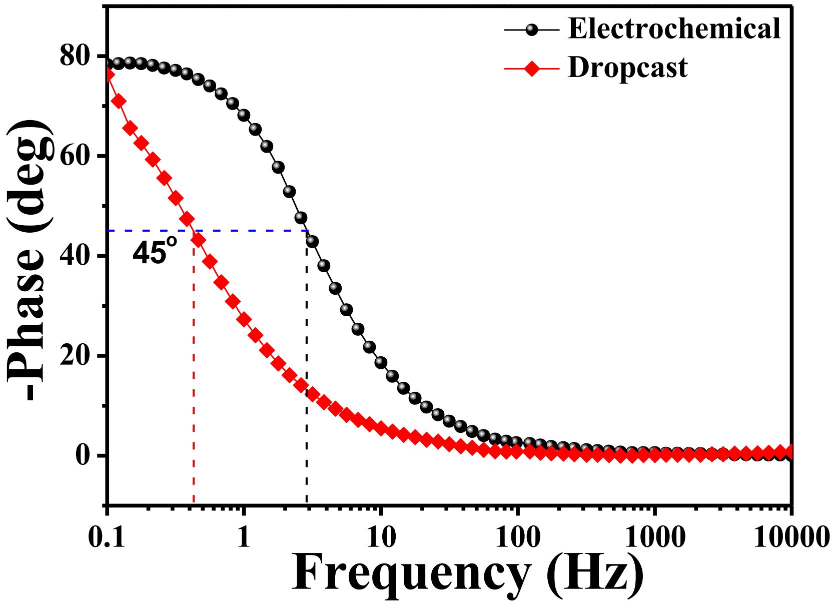

Fig. S8. Bode plot of the pErGO and the dropcast rGO materials.

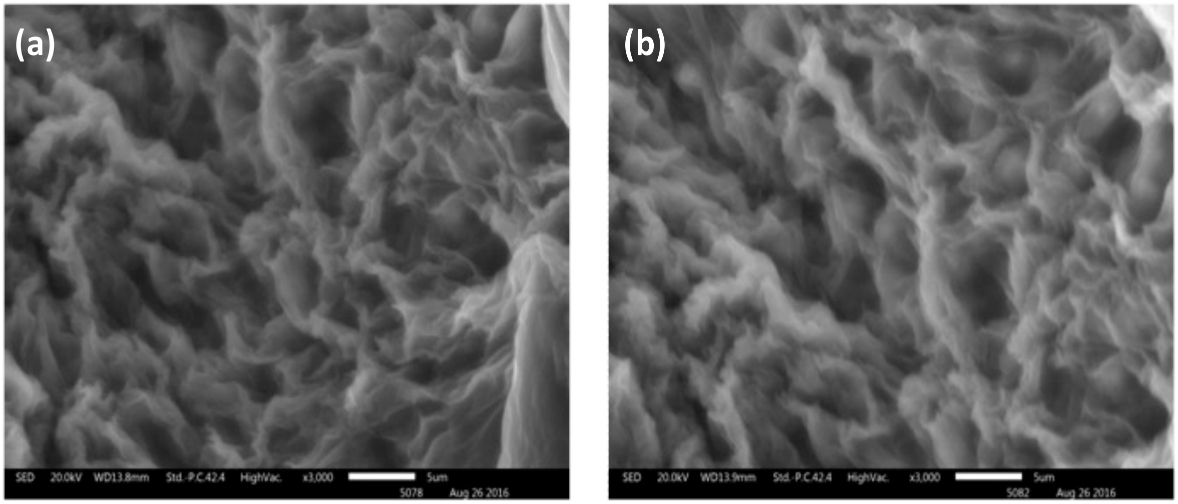

Fig. S9. SEM images of the fabricated pErGO@Cuf/Cu-wire electrode surface before (a) and after 5000 cycles (b). Scale bar: 5 μm.

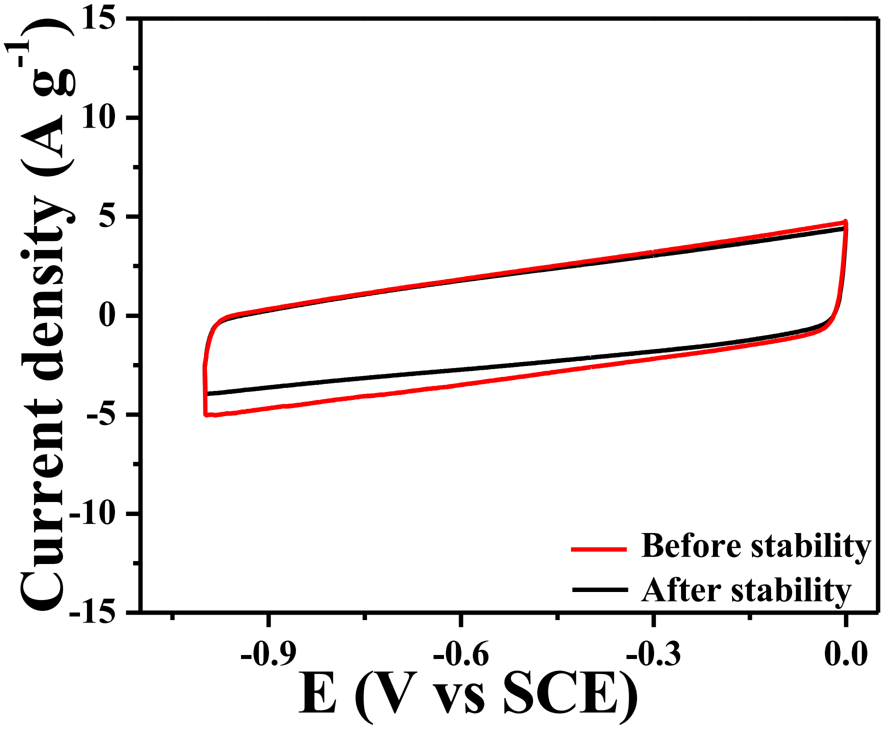

Fig. S10. CV recorded at before and after 5000 cycles of charge-discharge at 5 A/g. Scan rate: 100 mV/s.

Table S1. Summarized equivalent circuit parameters from fitted Nyquist plot

	ESR (Ω)	R_{CT} (Ω)	C_{DL} (F g^{-1})	W_o		C_F (F g^{-1})
				A (Ω s^{-n})	n	
pErGO	29	18	4E-06	495	0.43	0.00048

Table S2. Summarized frequency parameters and relaxation time constants from the Bode plot at different bending angles

Device angle (°)	Knee frequency (f_0) (Hz.)	Relaxation time constant (τ_0) (ms)
180	2.87	348
135	2.29	435
90	1.86	533
45	1.51	664

Table S3. Percentage capacitance retention after 5000 cycles at different bending angle

Device angle (°)	% C_{SP} retention after 5000 cycles
180	94.50
135	93.78
90	92.29
45	91.84

References

(1) Dey, R. S.; Hjuler, H. A.; Chi, Q. *J. Mater. Chem. A* **2015**, *3* (12), 6324–6329.

(2) Kovtyukhova, N. I.; Ollivier, P. J.; Martin, B. R.; Mallouk, T. E.; Chizhik, S. a.; Buzaneva, E. V.; Gorchinskiy, A. D. *Chem. Mater.* **1999**, *11* (3), 771–778.

(3) Zhu, N.; Han, S.; Gan, S.; Ulstrup, J.; Chi, Q. *Adv. Funct. Mater.* **2013**, *23* (42), 5297–5306.

(4) Yang, P.; Mai, W. *Nano Energy* **2014**, *8*, 274–290.

(5) Shao, Y.; El-Kady, M. F.; Wang, L. J.; Zhang, Q.; Li, Y.; Wang, H.; Mousavi, M. F.; Kaner, R. B. *Chem. Soc. Rev.* **2015**, *44*, 3639–3665.

(6) Ramadoss, A.; Kang, K.-N.; Ahn, H.-J.; Kim, S.-I.; Ryu, S.-T.; Jang, J.-H. *J. Mater. Chem. A* **2016**, *4* (13), 4718–4727.

(7) Liu, Y.; Weng, B.; Razal, J. M.; Xu, Q.; Zhao, C.; Hou, Y.; Seyedin, S.; Jalili, R.; Wallace, G. G.; Chen, J. *Sci. Rep.* **2015**, *5* (October), 17045.

(8) Zhang, L. L.; Zhao, X. S. *Chem. Soc. Rev.* **2009**, *38* (9), 2520–2531.

(9) Yu, D.; Goh, K.; Wang, H.; Wei, L.; Jiang, W.; Zhang, Q.; Dai, L.; Chen, Y. *Nat. Nanotechnol.* **2014**, *9* (7), 555–562.
